# Trends in estimated intramammary antimicrobial usage in the Irish dairy industry from 2003 to 2019

**DOI:** 10.3168/jdsc.2021-0081

**Published:** 2021-07-01

**Authors:** Catherine I. McAloon, Finola McCoy, Simon J. More

**Affiliations:** 1 UCD School of Veterinary Medicine, University College Dublin, Belfield, Dublin D04 V1W8, Ireland; 2 Animal Health Ireland, 4-5 The Archways, Carrick on Shannon, Co. Leitrim, N41 WN27, Ireland

## Abstract

The objective of this study is to update earlier work on intramammary (IM) antimicrobial (AM) usage in Ireland. There is a need to measure AM usage in food-producing animals given increasing societal concerns about AM resistance as well as recent regulatory changes that dictate changes in how AM are used in food-producing animals and how AM sales and usage are recorded. National sales data were collected and used in this analysis. Sales of the number of IM AM tubes and amount of active ingredient sold were analyzed each year by product type [in-lactation (LC) therapy and dry cow (DC) therapy] and classification system (World Health Organization and more recent European Medicine Agency). Descriptive trends in estimated IM AM use are presented, including defined course dose (DCDvet; a technical unit for on-farm usage). There has been a decrease in estimated on-farm usage of IM AM during lactation, from 0.48 DCDvet/cow per year in 2015 to 0.43 DCDvet/cow per year in 2019. Almost all LC therapies sold include critically important AM (CIA), with 98% of the total DCDvet administered for LC therapy in 2019 containing at least 1 CIA. There has been a slow increase in tubes containing at least 1 highest priority CIA in LC therapies, from 0.01 DCDvet/cow per year in 2003, accounting for 2% of the total DCDvet administered for LC therapy, to 0.03 DCDvet/cow per year in 2019, accounting for 7% of the total DCDvet administered for LC use. The estimated usage of IM AM DC therapy has decreased from 1.09 DCDvet/cow per year in 2015 to 0.95 DCDvet/cow per year in 2019. In the last 5 yr, more than 40% of the total DC DCDvet administered contained at least 1 CIA, and there has been an increase in recent years in the percentage of the total DC DCDvet administered that contains at least 1 highest priority CIA, driven mainly by use of fourth-generation cephalosporin. This work provides further insights into IM AM usage in Ireland and highlights some important areas for attention, including availability of farm-level usage data, prescribing practices, and usage of important AM.

## Highlights

• Blanket use of dry cow AM therapy is still very common in the Republic of Ireland.
• There is evidence of ongoing use of highest priority critically important AM.
• We identify key knowledge gaps and areas for urgent review in AM prescribing practices and acquiring prescriber- and user-level data.

## Graphical Abstract

**Figure:**
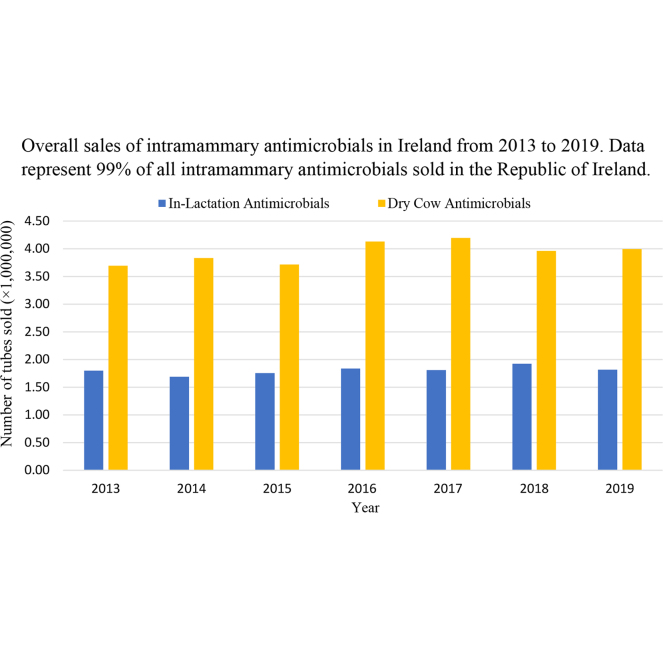
**Summary:** Understanding antimicrobial (AM) usage is key to safely reducing AM use in food-producing animals as part of addressing the global public health threat of AM resistance. We estimated intramammary AM usage from national sales data for the period 2003 to 2019, updating an earlier work. Descriptive trends in AM use are presented. We observed some reduction in dry cow AM use when accounting for cow numbers; however, use of blanket dry cow therapy is still widespread. In addition, there is evidence of ongoing use of highest priority critically important AM. This study provides objective evidence in support of efforts to direct and prioritize urgent changes in AM prescribing and usage as well as securing farm- and prescriber-level data in Ireland.

Antimicrobial (**AM**) resistance is an increasing threat to international public health, and a global action plan has been developed by the World Health Organization (**WHO**; WHO, 2015). Further, given the complex relationship between AM resistance in animals, humans, and the environment, a coordinated One Health approach to AM resistance is being taken (McEwen and Collignon, 2018). It is recognized that AM usage in both human and veterinary medicine accelerates the development of AM resistance (O'Neill, 2016), and although only limited data are available internationally, it is estimated that substantially more than 50% of AM by volume are used in food animals (Collignon et al., 2016). Although knowledge of the relative contribution of AM usage in food animals to human AM resistance is imperfect, there are examples of linkages between AM resistance in food animals and humans through the acquisition of resistant bacteria or, more importantly, through the spread of resistance genes (Collignon et al., 2016). It has been demonstrated that reducing AM usage in food-producing animals is linked with reducing AM resistance, and it is biologically plausible that these efforts are linked with reducing AM resistance in humans (Scott et al., 2018).

There are now multiple initiatives by both international organizations and national governments to encourage prudent usage of AM in food animals. The World Organization for Animal Health has developed supporting resources, including strategies on the prudent use of AM in food animals, intergovernmental standards on AM resistance, and national measurement of AM usage (OIE, 2016). The European Union (**EU**) recently introduced new legislation on veterinary medicines (European Union, 2019) that seeks, among other aims, to reduce AM use in food animals, particularly prophylactic use. The legislation also outlines requirements for harmonized collection of data on AM usage in farm animals as well as restrictions on AM that are designated as important for use in humans (More, 2020). This new legislation will have major implications on AM prescribing and use in the EU.

Data on AM usage in farm animal production are needed to objectively monitor progress. With these data, it is possible to conduct benchmarking (nationally, by sector, by prescriber, and at farm level) and to allow critical evaluation of usage patterns for both domestic use and international comparison. Although usage data at the level of the farm and veterinary prescriber are available in several countries, including Denmark and the Netherlands, data are more frequently available (and monitored) at a national level—for example, from AM sales (Grave et al., 2014; Merle and Meyer-Kühling, 2020). With the exception of targeted research studies, such as that reported by Earley et al. (2019), farm or veterinary prescriber data on AM usage are not currently available in Ireland. Using sales data, More et al. (2017) presented detailed insights into estimated intramammary (**IM**) AM usage in Ireland from 2003 to 2015.

Following the introduction in 2010 of CellCheck, the Irish national mastitis control program led by Animal Health Ireland, there have been substantial improvements in national milk quality as measured by bulk tank SCC. Based on bulk tank SCC data, in 2019, 66% of herds in Ireland had an annual unadjusted geometric mean SCC <200,000 cells/mL compared with 39% of herds in 2013. Since 2015, there have been many changes to the Irish dairy industry, with potential implications for AM usage including a major change in the demographic of the Irish dairy industry with rapid expansion, increasing global concern (and action) with respect to AM resistance, and EU legislation prohibiting blanket dry cow therapy from 2022. The Irish dairy industry has undergone rapid expansion following the abolition of EU milk quotas in 2015, including an 18.5% increase in the national milk output in the first year after quota abolition (Läpple and Sirr, 2019). The purpose of the current study is to update earlier work (More et al., 2017), describing trends in IM AM usage in the Irish dairy industry from 2003 to 2019.

The AM sales data for Ireland from 2003 to 2019 were obtained from 2 sources representing all relevant sources of IM AM sold in Ireland during this period. Sales data for 2003 to 2019 were obtained from Kynetec (Newbury, UK), an international market research company that gathers data on all IM AM sales conducted through the main drug wholesalers. These data also contained updated sales figures for the period 2003 to 2015 that had previously been analyzed by More et al. (2017). In addition, sales data for 2011 to 2019 were provided by 1 manufacturer, and sales data for 2013 to 2019 were provided by another 2 distributors of IM AM, whose sales data are not supplied directly to Kynetec. The data sets were subsequently reconciled to avoid any data duplication. The population of interest was all dairy cows in Ireland.

Dairy cow numbers were obtained from Eurostat (2021). Dairy cow numbers remained relatively constant between 2010 and 2013 (1.01 million and 1.08 million, respectively) but increased steadily from 2014 onward. Dairy cow numbers taken from Eurostat were 1.13, 1.24, 1.30, 1.34, 1.37, and 1.43 million cows for the years 2014 through 2019, respectively.

Two different AM classification systems were considered. The WHO classification reflects the importance of different AM groups for human medicine (WHO, 2018): very important AM, critically important AM (**CIA**), or highest priority CIA (**HP CIA**). The European Medicines Agency (**EMA**) classification categorizes antibiotics for prudent and responsible use in animals (EMA, 2019). Taking both AM classification systems into consideration, the following explanation is relevant to IM AM usage in Ireland:

• EMA category A (“avoid”): none of these drug groups are used in IM AM in cattle.
• EMA category B (“restrict”): includes third- and fourth-generation cephalosporins, which are classed as HP CIA in the WHO classification system.
• EMA category C (“caution”): includes macrolides, which under the WHO classification are classed as HP CIA drugs. This category also includes drugs such as aminoglycosides and moderate- and broad-spectrum penicillins (including aminopenicillins with β-lactamase inhibitors) that are classified as CIA by the WHO. In addition, first- and second-generation cephalosporins that are classed as not CIA or highly important by the WHO are labeled as category C under the new EMA regulation.
• EMA category D (“prudence”): these include drugs such as lincosamides, penicillins (antistaphylococcal penicillin, including cloxacillin and nafcillin), sulfonamides, dihydrofolate reductase inhibitors, and tetracyclines. These drugs are all classed as not CIA or highly important by the WHO. From 2018 onward, natural narrow-spectrum penicillins, including benethamine penicillin, penethamate hydriodide, and procaine benzylpenicillin, are also classified as not CIA or highly important (previously classified as CIA).

The numbers of tubes sold each year by product type [in-lactation (**LC**) therapy or dry cow (**DC**) therapy] and by WHO and EMA classification were calculated. We also calculated the quantity of AM sold (based on weight of active substance) that were CIA or HP CIA and the number of LC or DC tubes that contained either at least 1 or no CIA and at least 1 or no HP CIA. Sales data for internal teat sealant were not analyzed in this study.

As described previously (More et al., 2017), on-farm usage was estimated using the technical units daily defined dose (DDDvet) and defined course dose (**DCDvet**) per cow per year (and per 1,000 cow-days). In these calculations and to calculate the number of cows eligible for DC treatment, mean intercalving intervals of 391 d from 2003 to 2015 inclusive and 388, 390, 387, and 390 for the years 2016 to 2019, respectively, were used based on data from the Irish Cattle Breeding Federation for herds with more than 30 calvings annually (ICBF, 2020). The mean length of the dry period was assumed to be 60 d, and mean annual replacement rates of 21% for 2003 to 2015 inclusive and 22, 21, 21, and 20% for the years 2016 to 2019, respectively, were used (ICBF, 2020). The number of cows eligible for DC therapy was calculated each year as follows using data from the Irish Cattle Breeding Federation:

number of dairy cows in the country × (1 − annual replacement rate) × 365/mean intercalving interval.

Nulliparous heifers or cows at the end of their lactation were not assumed to be eligible for DC therapy. The 2003 to 2010 data were assumed to represent 85% of all IM sales, the 2011 and 2012 data to represent 90%, and the 2013 to 2019 data to represent 99%. These assumptions were determined following discussion with data providers based on all verified distribution routes for IM AM into Ireland during these periods. Data management and analysis were conducted in Microsoft Excel (Microsoft Corp.).

We report a decrease in estimated LC IM AM usage during 2003 to 2019, from 0.66 to 0.43 DCDvet/cow per year (Table 1; Figure 1). This decrease occurred throughout this period, including from 2015 (0.48) to 2019 (0.43). This is also reflected in number of tubes (Figure 2) and quantity of active substance, although this information does not account for the changes in cow numbers that occurred during this period. Almost all LC therapy included CIA, and there was a slow increase in tubes containing at least 1 HP CIA across the period (Table 1). In 2019, 7% of the total LC DCDvet administered was from tubes containing at least 1 HP CIA, either third- or fourth-generation cephalosporins, compared with 8% in 2015 and 2% in 2003 (Table 1).

**Table 1:** Annual intramammary antimicrobial usage in Ireland from 2003 to 2019^1^

Antimicrobial usage	2003	2004	2005	2006	2007	2008	2009	2010	2011	2012	2013	2014	2015	2016	2017	2018	2019
In-lactation therapy																	
All tubes																	
DDDvet/cow per year	1.99	1.80	1.98	1.81	2.08	2.10	1.89	1.94	1.99	2.07	1.68	1.51	1.43	1.43	1.36	1.42	1.29
Tubes with ≥1 CIA																	
DDDvet/cow per year	1.96	1.77	1.95	1.79	1.99	2.07	1.88	1.87	1.91	2.00	1.61	1.45	1.38	1.38	1.34	1.39	1.26
% of total DDDvet	98	98	99	99	96	99	99	96	96	97	96	96	97	96	98	98	98
Tubes with ≥1 HP CIA																	
DDDvet/cow per year	0.03	0.04	0.07	0.06	0.07	0.08	0.08	0.14	0.14	0.14	0.13	0.10	0.11	0.10	0.10	0.11	0.09
% of total DDDvet	2	2	3	3	3	4	4	7	7	7	8	6	8	7	7	8	7
All tubes																	
DCDvet/cow per year	0.66	0.60	0.66	0.60	0.69	0.70	0.63	0.65	0.66	0.69	0.56	0.50	0.48	0.48	0.45	0.47	0.43
Tubes with ≥1 CIA																	
DCDvet/cow per year	0.65	0.59	0.65	0.60	0.66	0.69	0.63	0.59	0.64	0.61	0.54	0.48	0.46	0.46	0.45	0.46	0.42
% of total DCDvet	99	99	99	99	96	99	99	91	96	88	96	96	97	96	98	98	98
Tubes with ≥1 HP CIA																	
DCDvet/cow per year	0.01	0.01	0.02	0.02	0.02	0.03	0.03	0.05	0.05	0.05	0.04	0.03	0.04	0.03	0.03	0.04	0.03
% of total DCDvet	2	2	3	3	3	4	4	7	7	7	8	6	8	7	7	8	7
Dry cow therapy																	
All tubes																	
DCDvet/cow per year	0.79	0.70	0.77	0.68	0.93	0.95	0.92	1.01	1.37	1.27	1.18	1.18	1.09	1.10	1.07	0.98	0.95
Tubes with ≥1 CIA																	
DCDvet/cow per year	0.39	0.34	0.35	0.30	0.42	0.38	0.32	0.36	0.63	0.59	0.55	0.55	0.47	0.52	0.49	0.44	0.39
% of total DCDvet	49	48	46	44	45	39	35	36	46	47	47	47	43	48	46	45	41
Tubes with ≥1 HP CIA																	
DCDvet/cow per year	0.00	0.00	0.00	0.00	0.00	0.00	0.00	0.02	0.06	0.05	0.05	0.05	0.07	0.07	0.08	0.09	0.10
% of total DCDvet	0	0	0	0	0	0	0	1	4	4	5	4	6	6	8	10	11

1 Antimicrobial usage is expressed as defined daily dose (DDDvet, for in-lactation therapy) or defined course dose (DCDvet, for in-lactation and dry cow therapy). Antimicrobial usage of tubes containing either critically important antimicrobials (CIA) or highest priority CIA (HP CIA) is also presented, expressed either as technical units (DDDvet or DCDvet) or the % of total DDDvet or DCDvet administered in the year of interest.

**Figure 1:**
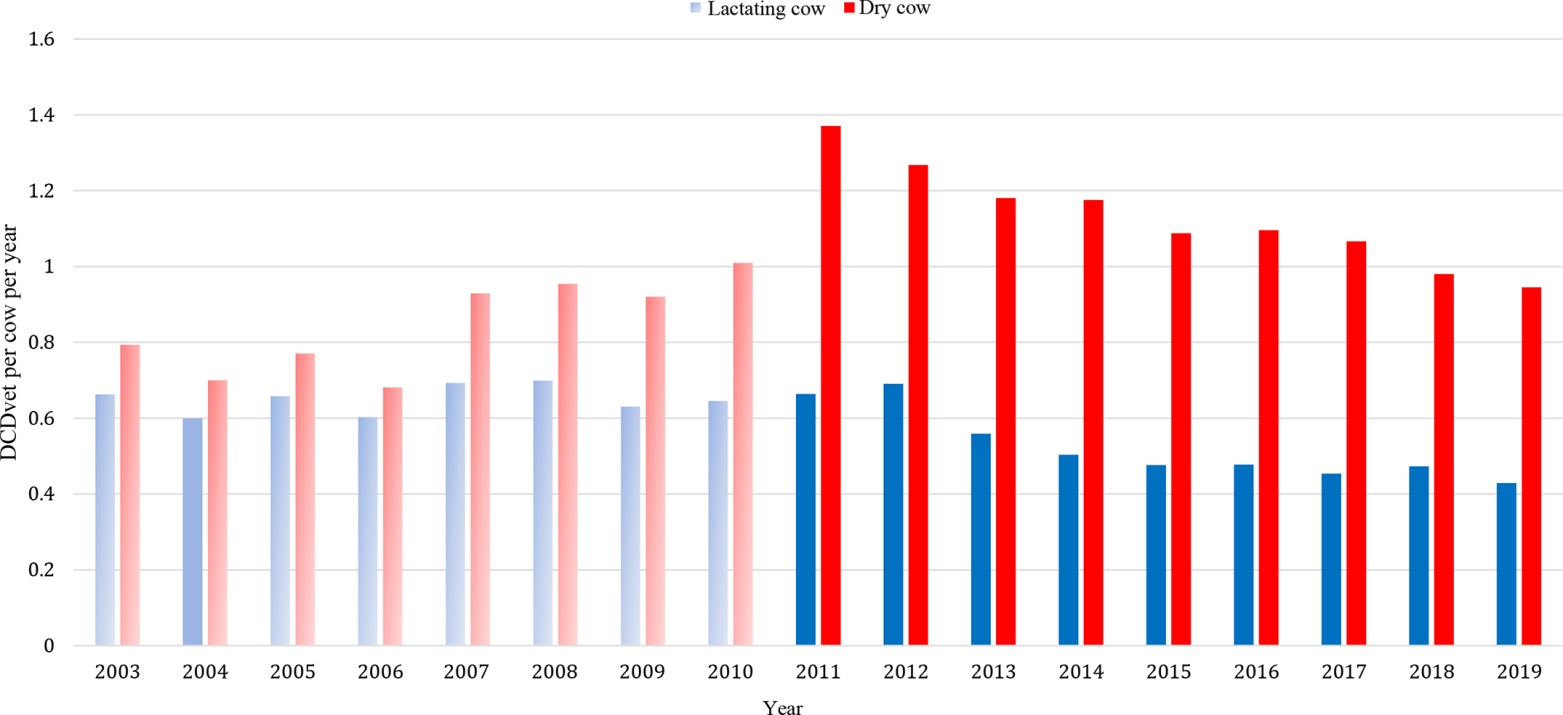
Estimated on-farm intramammary antimicrobial usage for in-lactation and dry cow therapy in Ireland from 2003 to 2019, expressed as defined course dose (DCDvet). Data are based on sales data collated by Kynetec (Newbury, UK) and 3 other individual suppliers, which are assumed to represent 99% of sales data from 2013 onward. Light-shaded bars represent periods where data were collected from Kynetec only. Dark-shaded bars represent periods where multiple data sources were available.

**Figure 2:**
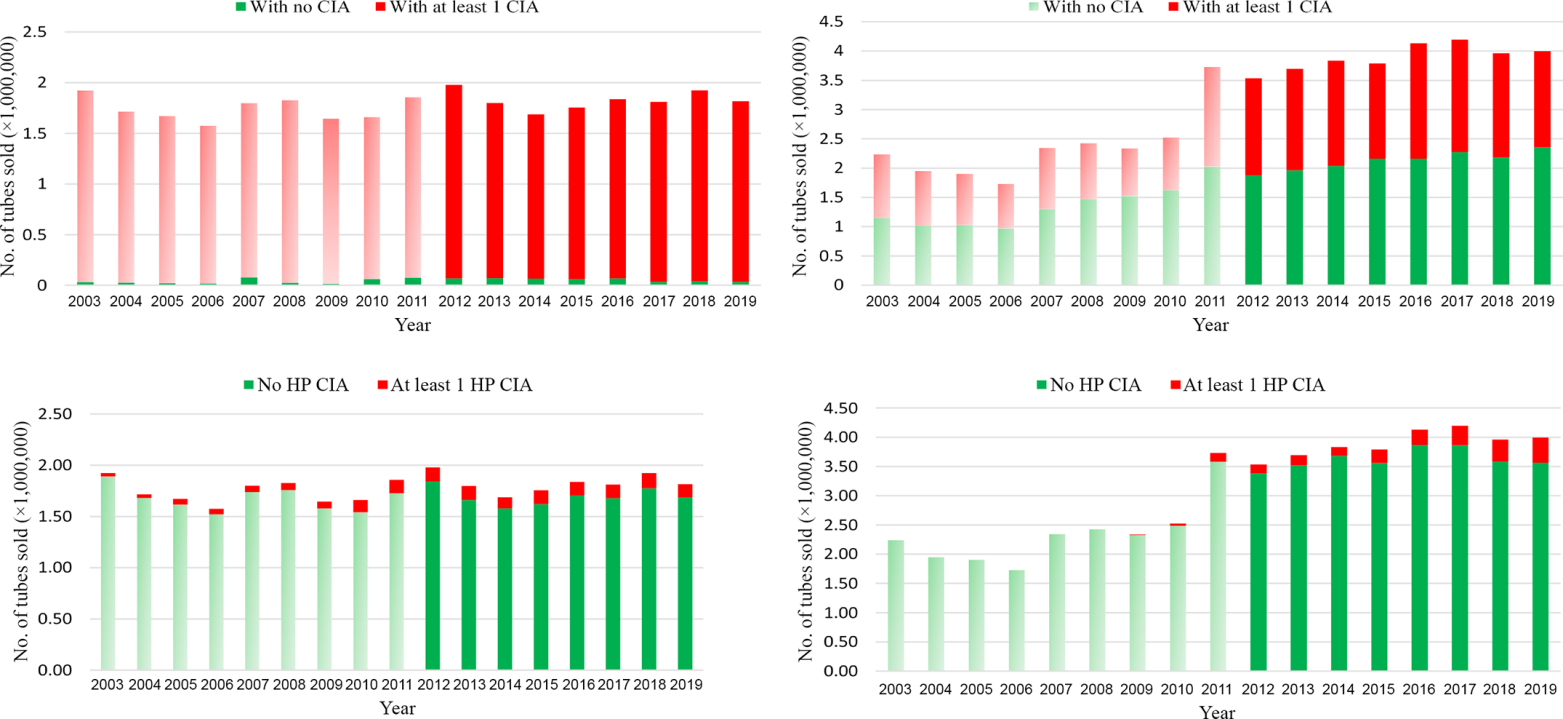
Number of intramammary antimicrobial tubes sold annually in Ireland from 2003 to 2019 for in-lactation (left) and dry cow (right) therapy. The top graphs present the number of tubes containing no or at least 1 critically important antimicrobial (CIA), and the bottom graphs present the number of tubes containing no or at least 1 highest priority CIA (HP CIA). These data are based on sales data collated by Kynetec (Newbury, UK) and 3 other individual suppliers, which are assumed to represent 99% of sales data from 2013 onward. Light-shaded bars represent periods where data were collected from Kynetec only. Dark-shaded bars represent periods where multiple data sources were available.

We also report a decrease in estimated DC IM AM usage during 2015 to 2019, from 1.09 to 0.95 DCDvet/cow per year. In the preceding period, particularly 2003 to 2011, there had been a substantial increase in DC IM AM usage, from 0.79 to 1.37 DCDvet/cow per year, as reported previously (More et al., 2017). Therefore, the estimated coverage of DC therapy in 2019 was 95%. These broad changes are also reflected in the number of tubes sold (Figure 1) and quantity of active substance, although this information does not account for changes in cow numbers. During 2003 to 2019, approximately half of the total DC DCDvet administered was from tubes containing at least 1 CIA (Table 1), ranging from 49% in 2003 to 41% in 2019. In recent years, there has been a sharp increase in the percentage of the total DC DCDvet containing at least 1 HP CIA (a fourth-generation cephalosporin), from 1% in 2010 to 6% in 2015 and 11% in 2019 (Table 1).

This study reports some positive improvement in usage, particularly in terms of overall AM usage during lactation and at drying off. The decrease in LC usage occurred concurrently with the rollout of the national CellCheck program and may reflect national improvement in udder health. Overall, LC usage can be favorably compared with comparator countries. A UK study (Humphry et al., 2021) reported a DCDvet for IM AM during lactation of 0.59/cow per year, which is calculated from sales data. Another UK study (Hyde et al., 2017) reported 0.82 DCDvet from 2017 data. Further, there was a gradual reduction in DC AM usage between 2015 and 2019; usage in 2019, the most recent year of this study, was 0.95 DCDvet/cow per year. However, these figures suggest only a limited move toward selective DC therapy, noting the legal requirement with EU Regulation 2019/6 (European Union, 2019) to cease prophylactic AM usage beginning January 28, 2022. Hyde et al. (2017) reported DC IM usage as 0.68 DCDvet/cow per year from 2017 data in the United Kingdom. An Austrian study reported 0.86 DCDvet/cow per year for DC therapy on conventional Austrian farms based on data from 2015 to 2016 (Firth et al., 2019). Under the auspices of CellCheck, there is a key focus on the prudent use of AM, including advice and resources in support of selective DC therapy on Irish farms (Animal Health Ireland, 2018). Given the urgent need to reduce the prophylactic use of AM, the observed reduction in DC usage is an important demonstration of early positive progress for the industry; however, continued progress is needed.

This study highlights ongoing heightened concerns about the type of AM sold for IM use, both LC and DC. Aminoglycosides and first-generation cephalosporins, which are both classified as category C (“caution”) under recent EMA (2019) guidelines, were among the most common active substances in both LC and DC AM sold in Ireland from 2015 to 2019. Further, there is evidence of ongoing usage of third- and fourth-generation cephalosporins in LC and of increasing usage of fourth-generation cephalosporins in DC, which are classed as HP CIA by WHO and category B (“restrict”) by EMA due to their importance for human health. Third- and fourth-generation cephalosporins should not be used in animal health unless a suitable alternative is unavailable and should be used only based on results of culture and susceptibility diagnostics proving they are the only option (EMA, 2019). There is anecdotal evidence of inadequate use of suitable diagnostics to support evidence-based, prudent prescribing of both LC and DC AM.

Under national legislation (European Communities, 2007), 2 prescribing routes for IM AM are currently allowed in Ireland: routine prescribing directly from a veterinary practitioner, and schedule 8 prescribing via the milk purchaser. With the latter, Irish farmers may obtain IM AM under a specific program for prevention and control of mastitis outlined by the milk purchaser. Upon direction by a veterinary practitioner overseeing such a mastitis program on behalf of the milk purchaser, IM AM can be prescribed to the farmer without attending the farm. We had intended to also evaluate usage by prescribing route, as done previously (More et al., 2017); however, this was not possible due to concerns about the quality and completeness of schedule 8 prescribing data during the period of interest. Nonetheless, based on the data available, it is clear that schedule 8 prescribing was used in Ireland from 2015 to 2019. In our view, this prescribing route is unlikely to provide the veterinary oversight necessary to support prudent prescription decision making on the basis of a detailed, on-farm understanding of mastitis and farm management. As a result of societal concerns and recent legislative change, we recommend an urgent review of overall prescribing practices for IM AM in the context of responsible AM stewardship.

This study was conducted using national AM sales data, as previously done (More et al., 2017), in the absence of farm-level AM usage data. This raises several concerns. First, there are inherent difficulties in seeking to link AM sales with specific usage patterns in farmed animal populations, although these concerns are lessened given the specialized nature of the IM products. In addition, sales data are equivalent to usage data only if AM products are being given at the correct dose and for the appropriate course (Mills et al., 2018). Second, higher resolution data, to the level of the farm and prescriber, are needed to facilitate benchmarking, as detailed by Collineau et al. (2017). The strategy of farm and prescriber objective measurement and benchmarking, linked with actual usage, has proven critical in countries such as Denmark and the Netherlands where AM usage has substantially decreased (Dorado-García et al., 2016; Andersen and Hald, 2017; DANMAP, 2021). These lessons are also relevant to Ireland.

This study has several limitations, and the study results need to be interpreted with care. Despite capturing all verified sources of IM AM (both direct and indirect) in Ireland from 2013 to 2019, we acknowledge other minor exceptions that are not captured, such as the importation by a merchant or wholesaler of a dual-licensed product from the United Kingdom into the Republic of Ireland. For this reason, these data are assumed to represent 99% (rather than 100%) of all sales. By necessity, several assumptions have been made relating to the number of cows eligible for DC therapy. We acknowledge the potential for retained unused product and for off-label usage of IM products in heifers or suckler animals or to treat conditions other than mastitis. In these circumstances, the usage estimates presented in this paper are overestimates of actual IM usage in the Irish dairy industry. For reasons outlined previously, it was not possible to conduct analyses such as prescribing route by WHO classification. Kynetec provided some data for 2003 to 2015 that were updated to reflect adjustments due to product returns, leading to several altered data points from the earlier data set used by More et al. (2017). Most internal teat sealants currently licensed in Ireland do not require a prescription for sale; therefore, the number of potential retail channels for these products is much greater than that for prescription products. For this reason, it is not currently possible to conduct any robust analysis of sales of internal teat sealants.

In conclusion, this update on the work of More et al. (2017) has added further detail to the quantification of AM usage in Ireland. This work, which is timely against the backdrop of the new EU medicines legislation, provides objective evidence in support of efforts to direct and prioritize urgent changes in AM prescribing and usage and to secure farm- and prescriber-level data in Ireland.
